# MicroRNA-29a Mitigates Osteoblast Senescence and Counteracts Bone Loss through Oxidation Resistance-1 Control of FoxO3 Methylation

**DOI:** 10.3390/antiox10081248

**Published:** 2021-08-04

**Authors:** Wei-Shiung Lian, Re-Wen Wu, Yu-Shan Chen, Jih-Yang Ko, Shao-Yu Wang, Holger Jahr, Feng-Sheng Wang

**Affiliations:** 1 Core Laboratory for Phenomics and Diagnostic, Department of Medical Research, College of Medicine, Chang Gung University, Kaohsiung Chang Gung Memorial Hospital, Kaohsiung 83301, Taiwan; lianws@gmail.com (W.-S.L.); ggyy58720240@gmail.com (Y.-S.C.); vip690221@gmail.com (S.-Y.W.); 2 Center for Mitochondrial Research and Medicine, Kaohsiung Chang Gung Memorial Hospital, Kaohsiung 83301, Taiwan; 3 Department of Orthopedic Surgery, College of Medicine, Chang Gung University, Kaohsiung Chang Gung Memorial Hospital, Kaohsiung 83301, Taiwan; ray4595@cgmh.org.tw (R.-W.W.); kojy@cgmh.org.tw (J.-Y.K.); 4 Department of Anatomy and Cell Biology, University Hospital RWTH Aachen, 52074 Aachen, Germany; h.jahr@masstrichtuniversity.nl; 5 Department of Orthopedic Surgery, Maastricht University Medical Center, 6229 ER Maastricht, The Netherlands

**Keywords:** microRNA-29a, senescence, osteoporosis, Oxr1, FoxO3, Dnmt3b

## Abstract

Senescent osteoblast overburden accelerates bone mass loss. Little is understood about microRNA control of oxidative stress and osteoblast senescence in osteoporosis. We revealed an association between microRNA-29a (*miR-29a*) loss, oxidative stress marker 8-hydroxydeoxyguanosine (8-OHdG), DNA hypermethylation marker 5-methylcystosine (5mC), and osteoblast senescence in human osteoporosis. *miR-29a* knockout mice showed low bone mass, sparse trabecular microstructure, and osteoblast senescence. *miR-29a* deletion exacerbated bone loss in old mice. Old *miR-29a* transgenic mice showed fewer osteoporosis signs, less 5mC, and less 8-OHdG formation than age-matched wild-type mice. *miR-29a* overexpression reversed age-induced senescence and osteogenesis loss in bone-marrow stromal cells. *miR-29a* promoted transcriptomic landscapes of redox reaction and forkhead box O (FoxO) pathways, preserving oxidation resistance protein-1 (*Oxr1*) and *FoxO3* in old mice. In vitro, *miR-29a* interrupted DNA methyltransferase 3b (Dnmt3b)-mediated *FoxO3* promoter methylation and senescence-associated β-galactosidase activity in aged osteoblasts. Dnmt3b inhibitor 5′-azacytosine, antioxidant N-acetylcysteine, or Oxr1 recombinant protein attenuated loss in *miR-29a* and *FoxO3* to mitigate oxidative stress, senescence, and mineralization matrix underproduction. Taken together, *miR-29a* promotes Oxr1, compromising oxidative stress and FoxO3 loss to delay osteoblast aging and bone loss. This study sheds light on a new antioxidation mechanism by which *miR-29a* protects against osteoblast aging and highlights the remedial effects of *miR-29a* on osteoporosis.

## 1. Introduction

Osteoporosis is a chronic skeletal disease with low bone mass and fragile microstructure [1], becoming a prominent risk factor of bone fracture-associated disability or premature death [2]. Osteoblast dysfunction, marrow adipocyte overgrowth, and osteoclast overactivation are notable features of osteoporotic bone [3]. Expanding evidence reveals that oxidative stress [4], mitochondrial dysfunction [5], and mesenchymal stem cell exhaustion [6] dysregulate bone microstructure integrity. In addition, an increased osteoblast senescence program, including upregulated p16^Ink4a^, p21^Cip1^ signaling, β-galactosidase activity, DNA damage, and senescence-associated secreted phenotypes [7]. inhibits bone formation activity during osteoporosis development [8].

Epigenetic alternation is one of the hallmarks of cellular aging, whereas a chronic dysregulation of the epigenetic program accelerates the development of age-related diseases [9]. Of epigenetic changes, methyl modification of cytosine in DNA into 5-methylcytosine (5mC), catalyzed by DNA methyltransferases (DNMT), represses promoter activity, inducing transcription repression to control tissue development and degeneration [10]. Increased 5mC formation is present in human age-related tissue dysfunction or tumorigenesis [11,12]. In skeletal tissue, DNA methylation and dysregulated DNMT influence osteogenesis and bone homeostasis. High DNA methylation signatures correlate with low bone mineral density [13] and postmenopausal osteoporosis [14]. DNMT3a mediates hypoxia mimetic-induced osteoblast dysfunction [15]. The DNMT3b-mediated DNA methylome impedes osteoblastogenesis of human mesenchymal progenitor cells from bone marrow [16]. Furthermore, oxidative stress induces DNMT3b-mediated *KLF5* hypermethylation, inhibiting osteogenic differentiation [17].

MicroRNAs (miR) bind to the 3′-untranslated region of mRNA targets, which interrupt protein translation and modulate plenty of biological activities [18]. These molecules affect bone-forming cell behavior or bone tissue integrity in aged skeleton. For example, *miR-219-5p* targets retinoic acid receptor-related orphan receptor beta, ameliorating osteogenesis loss [19]. The gain of *miR-195* function increases angiogenesis, progressively improving bone mass in aged mice [20]. *miR-31-5p* knockdown slows osteoclastic resorption and age-mediated bone loss [21]. MicroRNAs also interplay with epigenetic pathways, regulating the osteogenic lineage specification [22] and angiogenic activity [23] of mesenchymal stem cells. Little is known about microRNA signaling control oxidative stress, which inhibits osteoblast senescence and bone mass loss.

This study aimed to investigate whether *miR-29a* correlated with oxidative stress and osteoblast aging in human osteoporosis and examined if *miR-29a* knockout or overexpression affected osteoblast senescence and bone loss in old mice and characterized the epigenetic mechanism by which *miR-29a* controlled oxidative stress and senescence program in osteoblasts of osteoporotic skeleton.

## 2. Materials and Methods

### 2.1. Clinical Specimens

Studies on human bone biopsies were approved by the Chang Gung Medical Foundation Institutional Review Board (Affidavit #202000823B0). Informed consent was obtained from patients preoperatively. Thirteen patients with osteoporosis (5 males and 8 females) and 13 patients without osteoporosis (6 males and 7 females) who required lumbar spine decompression, fixation, discectomy, or vertebroplasty were enrolled in the osteoporosis group and control group, respectively. The BMD of the hip was quantified using dual-energy X-ray absorptiometry before surgery. Leftover bone biopsies and 10 mL peripheral blood were harvested.

### 2.2. miR-29a Knockout Mice

Animal experiments were approved by Institutional Animal Use and Care Committee, Kaohsiung Chang Gung Memorial Hospital (Affidavit #2014120401). The homologous arms of C57BL/B6 mouse *miR-29a* gene (Chromosome 6, 31029595-31049694) (RP23-173G8) of Bacterial Artificial Chromosome (BAC) library (BACPAC Genomics Resource Center; Emeryville, CA, USA) were cloned using PCR protocols. The *loxP* site and the neomycin (Neo) resistance cassette (*PL452*) were inserted into *PL253* plasmid (Addgene, Watertown, MA, USA). The *Neo* gene driven by *T7* promoter in mammalian cells was removed by Cre recombinase. The linearized BAC construct was transferred into embryonic stem cells of C57BL/6 mice to produce *miR-29a* chimera, which further crossed with *Sox2*-*Cre* mice (Jackson Laboratory, Bar Harbor, ME, USA) to breed miR-29aKO mice. The genotype of miR-29aKO mice was confirmed using customized primers (Supplementary Table S1) and PCR protocols. Mice were raised in a specific pathogen-free laboratory animal facility with feed and drinking water ad libitum.

### 2.3. Bone-Specific miR-29a Transgenic Mice

Osteoblast-specific *miR-29a* transgenic mice (C57L/B6; miR-29aTg), which carried osteocalcin (*Bgp*) promoter-driven human *miR-29a* precursor construct, were generated. In brief, linear constructs with osteocalcin (Bgp) promoter–human *miR-29a* precursor (506 bp) –bovine growth hormone (*BGH*) polyadenylation (*polyA*) were transfected into fertilized eggs from C57L/B6 mice through microinjection protocols. The micro-injected eggs were grafted into foster mother mice, as previously described [24]. Genotypes of littermates were characterized using PCR protocols and primers. Mice carrying the 506-bp gene coded human *miR-29a* precursor were considered miR-29aTg mice, whereas mice that did not carry the gene of interest were considered wild-type (WT) mice [24].

### 2.4. Age-Mediated Osteoporosis

Three-month-old and 9-month-old male miR-29aTg or WT mice were divided into young and old groups, respectively. To label bone mineralization, 10 mg/kg calcein was injected 9 days and 3 days before euthanasia. Peripheral blood was drawn and centrifuged to harvest serum. Femurs and tibiae specimens were dissected for subsequent experiments.

### 2.5. μCT Analysis of Bone Tissue and Marrow Adipose

The microstructure of femurs and tibiae was visualized using SkyScan 1176 μCT (Bruker, Belgium) and captured 200 radiographs (9-μm voxel size), as previously described [24]. BMD (g/cm^3^), trabecular volume (BV/TV, %), trabecular thickness (Tb.Th, mm), and trabecular number (Tb.N/mm) of proximal tibiae, were measured using SKYSCAN^®^ CT-Analysis software, according to the maker’s instructions. In a subset experiment, μCT radiography of marrow fat in tibiae was performed upon soaking in OsO_4_, as previously described [25]. The images of OsO_4_-soaked specimens were reconstructed and merged using the software. Marrow fat volume (mm^3^) and fat surface (mm^2^) were calculated automatically.

### 2.6. Biomechanical Test

Upon measuring the cross-section area of the middle region of femurs using μCT scanning protocols, the biomechanical properties, including breaking force (N/mm^2^) and energy (N.mm), of the middle region of femurs upon 3-point bending were quantified using SHIMADZU EZ-SX Material Test System (Shimadzu Corporation, Kyoto, Japan) with TRAPEZIVMX software, according to the maker’s instructions.

### 2.7. Histomorphometry and Immunohistochemistry

The calcein labeling in methyl acrylate (Polysciences Inc., Warrington, PA, USA)-embedded tibia sections was evaluated using a fluorescence microscope (Carl Zeiss, Oberkochen, Germany). Trabecular bone histology was evaluated using von Kossa staining kits (ab150687; Abcam, Cambridge, UK) and hematoxylin–eosin stain kits (Sigma-Aldrich, St Louis, MO, USA). Osteoclasts were probed using a tartrate-resistant acid phosphatase (TRAP) staining kit (B-Bridge International Inc., Mountain View, CA, USA). Trabecular volume (BV/TV, %), mineral acquisition rate (MAR, μm/day), and osteoclast number (Oc.N/mm) were quantified using Axio Image Analysis System (Carl Zeiss, Oberkochen, Germany). Six random fields in each section and 3 sections in each animal were selected for histomorphometry. Immunohistochemical staining for paraffine-embedded sections was performed using β-galactosidase (ab136776, Abcam, Cambridge, UK), p16^Ink4a^ (1E12E10, Invitrogen Thermo Fisher Scientific Inc., Waltham, MA, USA), 5-methylcytosine (5mC) (GT411, Invitrogen, Carlsbad, CA, USA), and 8-hydroxydeoxyguanosine (8-OHdG) (BS-1278R; Thermo Fisher Scientific Inc., Waltham, MA, USA). Immunostained osteoblasts in each high-power field (×400 magnification) were counted. Three random fields in each section and 3 sections in each tibia specimen were selected to quantify immunostained cells.

### 2.8. Ex Vivo Osteogenesis, Adipocyte, and Osteoclast Formation

Mesenchymal cells and macrophage precursor cells in bone marrow were isolated, as previously described [24]. Bone-marrow stromal cells (10^5^ cells/well, 24-well plates) were seeded in an osteogenic condition using StemProTM Osteogenesis Differentiation Kits (A1007201 Thermo Fisher Scientific Inc., Waltham, MA, USA) and in an adipogenic condition using StemProTM Adipogenic Differentiation Kits (A1007001) for 21 days and 15 days, respectively. Mineralized matrices and adipocytes were stained using von Kossa stain kits and Nile Red stain kits (Abcam, Cambridge, UK). Bone-marrow macrophage precursor cells (5 × 10^4^ cells/well, 48-well plates) were incubated in αMEM with 15 ng/mL M-CSF and 40 ng/mL RANKL (R&D Systems, Minneapolis, MN, USA) for 1 week. Osteoclasts were probed using TRAP stain kits. Von-Koss-stained mineralized matrices in each low-power field (x125 magnification) and Nile-red-stained adipocytes and TRAP-stained osteoclasts in each high-power field were measured using the Zeiss Image Analysis System.

### 2.9. Aged Osteoblast Cultures

Osteoblasts were multiple-passaged as an in vitro model of age-induced bone loss, as previously described [26,27,28]. Upon euthanasia, primary (P0) calvarial osteoblasts were isolated from 3-month-old mice and incubated in DMEM and 10% FBS. Primary osteoblasts (5 × 10^5^/well, 6-well plates) were incubated in medium for 3 days. After 10 passages, osteoblasts were designated to the aged group (P10). In some experiments, 10^5^ cells/well (24-well plates) were incubated in 100 nM 5-Aza-2′-deoxycystidine (5-aza; A3656, Sigma-Aldrich, St Louis, MO, USA) or 10 μM N-acetylcysteine (NAC; A9165, Sigma-Aldrich, St Louis, MO, USA) for 4 h and incubated in an osteogenic medium.

### 2.10. Oxidative Stress-Induced Osteoblast Senescence

Murine MC3T3-E1 preosteoblasts (2 × 10^4^ cells/48-well plate) were incubated in DMEM and 10% FBS with or without 300 µm H_2_O_2_ (H1009, Sigma-Aldrich, St Louis, MO, USA) [29] or 50 µM Oxr1 recombinant protein (MSB1070208; MyBiosource, San Diego, CA, USA) for 6 h and further incubated in osteogenic differentiation medium (DMEM, 10% FBS, 10 mM β-glcerophosphate and 50 mM ascorbate) for 15 days to investigate cellular senescence and mRNA expression.

### 2.11. Transfection of miR-29a Mimic and Antisense Oligonucleotide

A total of 10 nM *miR-29a* mimetic, antisense oligonucleotide, and scramble control (miRVana; Thermo Fischer Scientific Inc., Waltham, MA, USA) were added to Lipofectamine^TM^ 3000 (Invitrogen^TM^; Thermo Fischer Scientific Inc., Waltham, MA, USA) and transferred into P0 or P10 osteoblasts (5 × 10^5^ cells/well, 24-well plates).

### 2.12. Senescence-Association β-galactosidase (SA-β-gal) Staining

SA-β-gal activity in osteoblasts (10^3^ cells/slide) was probed using Senescence β-Galactosidase Staining Kits (#9860, Cell Signaling, Danvers, MA, USA). SA-β-gal-stained cells in each field, 3 fields in each slide, and 3 slides for each experiment were counted.

### 2.13. RT-PCR

Total RNA was extracted from WT and miR-29aTg osteoblasts using TRI Reagent™ Solution (Thermo Fisher Scientific Inc., Waltham, MA, USA). High-Capacity cDNA Reverse Transcription Kits (Thermo Fisher Scientific Inc., Waltham, MA, USA) were utilized to reversely transcribe 1 μg of total RNA. PCR was investigated using primers (Supplementary Table S1), 2× TaqMan^®^ Universal PCR Master Mix with ABI 7900 Detection System (Applied Biosystems, Foster City, CA, USA), and the threshold value for amplification reaction was probed. Equation 2^−ΔΔCt^ was used to calculate the fold change of mRNA expression.

### 2.14. Whole Genome Microarray Assay

A total of 10 μg of RNA was amplified using Amino Allyl MessageAmp II aRNA amplification kits (Ambion, Thermo Fisher Scientific Inc., Waltham, MA, USA) and coupled with CyDye using CyDye Post-Labeling Reactive Dye Packs (Cytiva, Marlborough, MA, USA), according to the makers’ instructions. Upon purification, the labeled aRNA was hybridized onto Mouse MOA2.0 whole-genome arrays (Phalanx Biotech Inc., San Diego, CA, USA). Data and clustering were processed using Rosetta Resolver Biosoftware^®^. Fold change of the gene expression log2 > 1 was considered differentially expressed. Gene ontology and KEGG pathway were clustered using Kobas bioinformatics engine (http://kobas.cbi.pku.edu.cn, accessed on 19 July 2021), as previously described [30].

### 2.15. Luciferase Activity Assay

Wild-type (5′-UUUUACCUAAUUACAGGUGCUAU-3′; ENST00000344505.4) or 5-bp mutant (5′-UUUUACCUAAUUACAGTAAACAU-3′) of the 3′-UTR of *Dnmt3b* was constructed into luciferase reporter *pCRII-TOPO II* vector (Invitrogen^TM^; Thermo Fischer Scientific Inc., Waltham, MA, USA). A total of 10 ng of luciferase vector and 10 ng Renilla luciferase reporter vector were mixed with LipofectamineTM 3000 Transfection Reagent (Invitrogen^TM^; Thermo Fischer Scientific Inc., Waltham, MA, USA) and transfected into osteoblasts (10^4^ cells/well, 96-well plate). The reporter cells were transfected into 30 nM scramble control, miR-29a mimetic, or antisense oligonucleotide. Fluorescent luciferase activity was probed using Dual Luciferase Detection Kits and normalized with Renilla luciferase activity.

### 2.16. Methylation-Specific PCR

Genomic DNA in 5 × 10^6^ osteoblasts was harvested using MagMAX™-96 DNA Multi-Sample Kits (4413021; Invitrogen, Danvers, MA, USA). Methylated DNA was probed using the EZ DNA Methylation™ Kit (Zymo Research, Irvine, CA, USA), according to the maker’s manuals. A total of 10 ng genomic DNA was mixed with 100 μL CT Conversion Reagent with bisulfite at 50 ℃ in the dark for 16 h and incubated at 4 ℃ for 10 min. Upon eluting through Zymo-Spin™ IC Column, the elute was washed with an M-Wash Buffer and mixed with a M-Desulphonation Buffer and an Elution Buffer. PCR analysis of the eluted DNA was performed using the ABI 7900 Detection System together with specific primers, methylated and unmethylated *FoxO3* promoter, and *miR-29a* promoter. PCR analysis of *GADPH* gene was performed to confirm the equal loading of elute DNA (Supplementary Table S1).

### 2.17. Immunoblotting

Lysates of 5 × 10^6^ osteoblasts were extracted using Mammalian Cell Lysis Kits (Sigma-Aldrich, St. Louis, MO, USA). Immunoblotting of 5mC, 5hmC, Foxo3, Dnmt3b, and actin levels in the lysates was performed using specific antibodies and SuperSignal™ Western Blotting Kits (Thermo Fisher Scientific Inc., Waltham, MA, USA).

### 2.18. Statistical Analysis

Differences of age, hip BMD, serum *miR-29a*, and immunostaining in the non-osteoporosis and osteoporosis groups were analyzed using a Wilcoxon test. The patients’ gender of both groups was analyzed using a Chi-square test. The investigations of WT and miR-29aKO mice were analyzed using a Student’s *t*-test. The investigations of young and old WT and miR-29aTg mice and in vitro models were analyzed using an ANOVA test and a Bonferroni post hoc test. *p* value < 0.05 resembled significant difference.

## 3. Results

### 3.1. miR-29a Loss, Senescent Osteoblast Overburden, and Oxidative Stress in Human Osteoporosis

**Figure 1:**
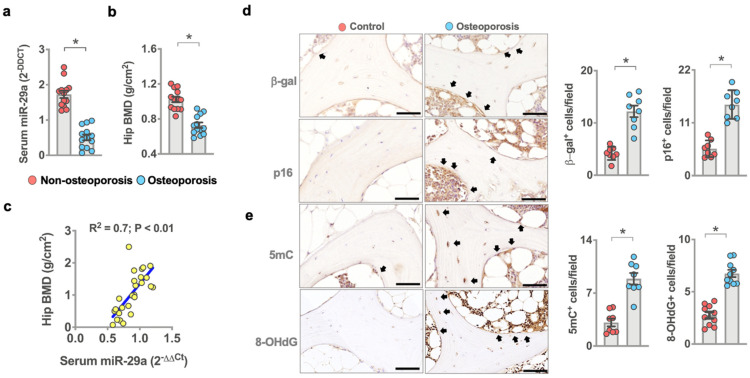
Analysis of *miR-29a*, osteoblast senescence, oxidative stress, and DNA methylation in human osteoporosis. Decreased serum *miR-29a* levels (**a**) and hip BMD (**b**) in the osteoporosis group. Serum *miR-29a* levels correlated with osteoporosis (**c**). Data are mean ± standard error calculated from 13 patients without osteoporosis and 13 patients with osteoporosis who required spine surgery. Osteoblasts in age-mediated osteoporotic bone showed strong β-galactosidase, p16 (**d**), 5mC, and 8-OHdG (**e**) immunostaining (arrows) (scale bar, 20 µm). Immunohistochemical data (mean ± standard error) are calculated from 7–8 bone biopsies. Significant difference (asterisks *) was analyzed using a Wilcoxon test.

We examined if oxidative stress or osteoblast senescence or *miR-29a* expression correlated with human osteoporosis. We collected bone biopsies and serum from 13 patients with osteoporosis (5 males and 8 females; 75.8 ± 1.1 years old) and 13 patients without osteoporosis (6 males and 7 females; 36.6 ± 1.9 years old) who required lumbar spine decompression, fixation, discectomy, or vertebroplasty. Serum *miR-29a* levels (Figure 1a) and bone mineral density of hips (Figure 1b) in patients with osteoporosis were less than in patients without osteoporosis. Serum *miR-29a* level significantly correlated with the development of osteoporosis (Figure 1c). Plenty of osteoblasts in osteoporotic bone specimens showed strong cellular senescence markers, including β-galactosidase and p16^Ink4a^ immunostaining (Figure 1d), as compared to the control group. Bone cells in the osteoporosis group also displayed strong DNA methylation marker 5-methylcytosine (5mC) immunoreaction and oxidative damage marker 8-hydroxydeoxyguanosine (8-OHdG) (Figure 1e) immunoreactivity, suggesting that DNA hypermethylation and oxidative stress were present in the development of osteoporosis.

### 3.2. miR-29a Deletion Induced Bone Loss and Inhibited Bone Formation Activity

**Figure 2:**
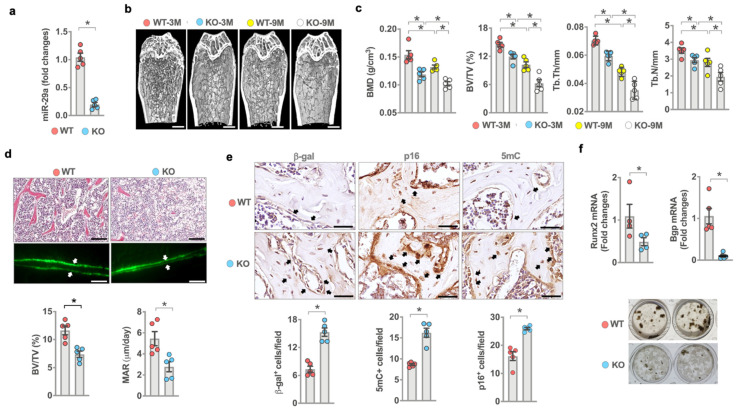
Analysis of *miR-29a* expression, osteoblast senescence, bone mass, and microstructure in miR-29a knockout mice. Low miR-29a expression in the bone tissue of 3-month-old miR-29aKO mice (**a**). μCT images showing sparse trabecular microstructure (**b**) (scale bar, 50 μm) and decreased BMD, BV/TV, Tb.Th, and Tb.N (**c**) in young and old miR-29aKO bone tissue. Significant differences of investigations (mean ± standard error, n = 5 mice) were analyzed using an ANOVA test and Bonferroni post hoc test. Three-month-old miR-29aKO bone tissue showed trabecular bone loss, marrow adiposis (scale bar, 20 μm), weak calcein labeling (scale bar, 40 μm), and decreased BV/TV and MAR (**d**). Osteoblasts in miR-29aKO bone tissue showed strong β-gal, p16, and 5mC immunostaining (**e**) (scale bar, 10 μm). miR-29a knockout reduced osteogenic gene expression and von-Kossa-stained mineralization matrix deposition of bone-marrow stromal cells (**f**). Significant differences (asterisks *) of investigations (mean ± standard error, *n* = 5–6 mice) were analyzed using the Wilcoxon test.

The findings of human bone specimens prompted us to investigate whether *miR-29a* loss affected bone tissue integrity. To this end, ubiquitous *miR-29a* knockout mice (miR-29a^loxp^-Sox2^Cre^; miR-29aKO) were generated by crossing *miR-29a^loxP^* mice with *Sox2^Cre^* mice. RT-PCR confirmed *miR-29a* loss in the bone tissue of 3-month-old miR-29a KO mice (Figure 2a). We divided 3- and 9-month-old mice into young and old mouse groups, respectively. Three-month-old miR-29aKO mice developed sparse trabecular bone microstructure (Figure 2b) together with deceased bone mineral density (BMD), trabecular volume (BV/TV), trabecular thickness (Tb.Th), and trabecular number (Tb.N) (Figure 2c). Of note, bone mass loss and porous trabecular architecture were worsened in 9-month-old miR-29aKO mice as compared to age-matched WT mice. Histological investigations revealed trabecular bone loss and marrow adiposis together with decreased trabecular volume (BV/TV) and low calcein-labeled mineral acquisition rate (MAR) in 3-month-old miR-29aKO skeleton (Figure 2d). Loss of *miR-29a* function upregulated senescence and DNA methylation in a high number of osteoblasts, which showed strong β-galactosidase, p16, and 5mC immunostaining (Figure 2e). Furthermore, bone-marrow mesenchymal cells in 3-month-old miR-29aKO mice showed less osteogenic differentiation potential, including decreased *Runx2* and *osteocalcin* (*Bgp*) expression and mineralized extracellular matrix underproduction (Figure 2f), as compared to age-matched WT mice.

### 3.3. Transgenic Overexpression of miR-29a Slowed Bone Loss in Old Mice

**Figure 3:**
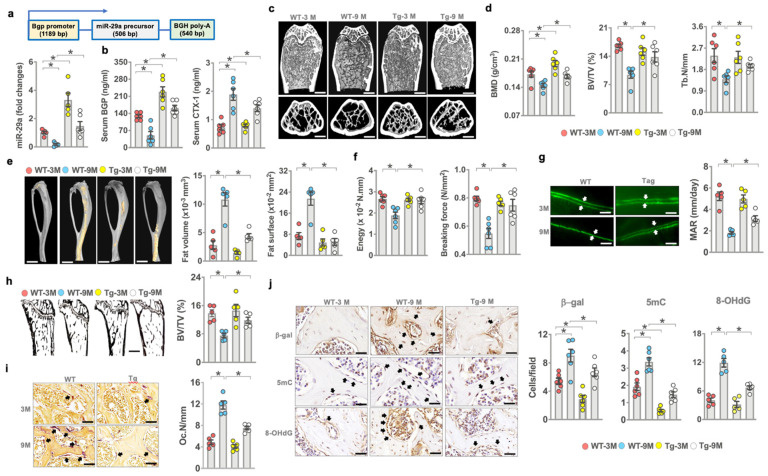
Effects of *miR-29a* overexpression on bone mass, mechanics, and histomorphometry of young and aged mice. Schematic drawing for the linear gene construct for the generation of osteoblast-specific miR-29aTg mice. miR-29a overexpression attenuated miR-29a loss in bone tissue (**a**) and serum Bgp and CTX-1 level alteration in old mice (**b**). μCT revealing sparse trabecular microstructure in old WT mice and well-woven bone architecture in old miR-29aTg mice (**c**); scale bar, 50 μm in upper panels; 90 μm in lower panels. miR-29a overexpression improved age-induced loss in BMD, BV/TV, and Tb.N (**d**). miR-29a compromised fatty marrow development (scale bar, 5 mm) (**e**) and biomechanics loss (**f**) and reversed mineral acquisition (scale bar, 40 μm) (**g**) and von-Koss-stained trabecular bone volume (scale bar, 40 μm) (**h**), as well as reduced TRAP-stained osteoclast formation (scale bar, 20 μm) (**i**). *miR-29a* overexpression attenuated β-galactosidase, 5mC, and 8-OHdG immunoreactivity (**j**); scale bar, 15 μm. The significant difference (asterisks *) of investigations (mean ± standard error, *n* = 5–6 mice) was analyzed using an ANOVA test and Bonferroni post hoc test.

Given that *miR-29a* loss accelerated bone mass loss and microstructure deterioration, we asked whether transgenic overexpression of *miR-29a* in bone tissue changed the skeletal integrity of old animals. We bred osteoblast-specific *miR-29a* transgenic mice under the control of the *Bgp* promoter (miR-29aTg) [24] (Figure 3a). We divided 3- and 9-month-old mice into young and old mouse groups, respectively. *miR-29a* expression in bone tissue (Figure 3a) and serum bone formation marker Bgp levels were downregulated in old WT animals, whereas serum resorption marker C-telopeptide of collagen I (CTX-1) levels were increased (Figure 3b). *miR-29a* loss and serum bone marker alteration were compromised in old miR-29aTg mice. Old WT mice developed sparse bone microstructure (Figure 3c) as evident from decreased BMD, BV/TV, and Tb.N (Figure 2d). Old miR-29aTg mice showed mild BMD loss and a well-connected trabecular bone network. Old WT bone tissue developed marrow adiposity as evidenced in increases in marrow fat volume and surface (Figure 3e). These animals had less bone biomechanical strength, including breaking force and energy (Figure 3f), than young WT mice. *miR-29a* overexpression compromised marrow fat overproduction and biomechanics loss in old mice.

Old WT bone tissue showed less von-Kossa-stained trabecular bone morphology (BV/TV; Figure 3g) and decreased bone mineral acquisition (MAR; Figure 3h) together with plenty of tartrate-resistant acid-phosphate-stained osteoclasts developed along trabecular bone (Oc.N/mm; Figure 3i) as compared to young WT mice. miR-29Tg bone revealed a mild loss in static and dynamic bone formation and osteoclast overburden. A high number of osteoblasts in old WT bone showed strong β-galactosidase, 5mC, and 8-OHdG immunoreactivity. Mild senescence, DNA methylation, and oxidative stress were present in osteoblasts in old miR-29aTg mice (Figure 3j).

### 3.4. miR-29a Attenuated Senescence and Osteogenesis Loss in Old Mice

**Figure 4:**
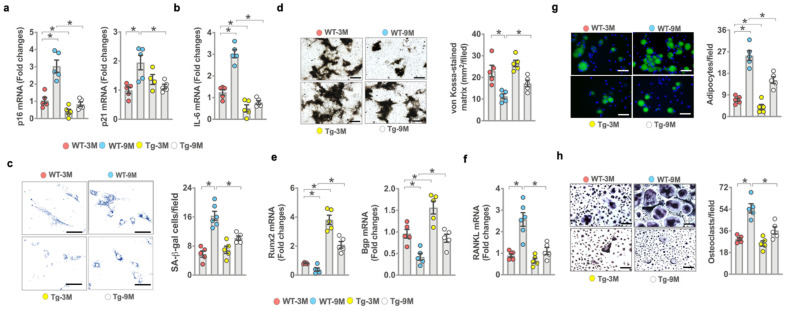
Effects of *miR-29a* overexpression on ex vivo osteogenic, adipogenic, and osteoclastogenic differentiation capacity. *miR-29a* repressed age-induced *p16*, *p21* (**a**), *IL-6* expression (**b**), and SA-β-galactosidase activity of primary bone-marrow mesenchymal cells (**c**); scale bar, 20 μm. miR-29a reversed age-induced loss in mineralized nodule formation (**d**) (scale bar, 40 μm) and (**e**) *Runx2* and *Bgp* expression (**e**) and decreased *RANKL* expression (**f**). *miR-29a* compromised (**g**) fluorescent Nile-red-stained adipocyte formation (scale bar, 20 μm) and (**h**) TRAP-stained osteoclast formation (scale bar 20 μm) in old mice. Significant difference (asterisks *) of investigations (mean ± standard error, *n* = 5) analyzed using an ANOVA test and a Bonferroni post hoc test.

Consistent with the analysis of senescent osteoblast overburden in osteoporotic bone, senescence program, including *p16^Ink4a^*, *p21^CIP^* expression (Figure 4a), inflammatory cytokine *IL-6* expression (Figure 4b), and SA-β-gal activity (Figure 4c) were significantly increased in bone-marrow mesenchymal cells of old WT mice. However, the osteogenesis of bone-marrow stromal cells as evidenced in von-Kossa-stained mineralized matrix production (Figure 4d), *Runx2*, and *Bgp* expression (Figure 4e) was reduced in old WT mice. Senescence program, inflammation, and osteogenesis loss were improved in old miR-29aTg mice. On the other hand, osteoclast factor *RANKL* expression (Figure 4f) and fluorescent Nile-red-stained adipocyte formation of bone-marrow stromal cells (Figure 4g), as well as TRAP-stained osteoclast formation of bone-marrow macrophage precursor cells (Figure 4h), were upregulated in old WT mice. The ex vivo adipocytic activity and osteoclastogenic differentiation capacity were compromised in old miR-29aTg mice.

### 3.5. miR-29a Controlled Redox Reaction, Oxidation Resistance-1, and FoxO3 Signaling

**Figure 5:**
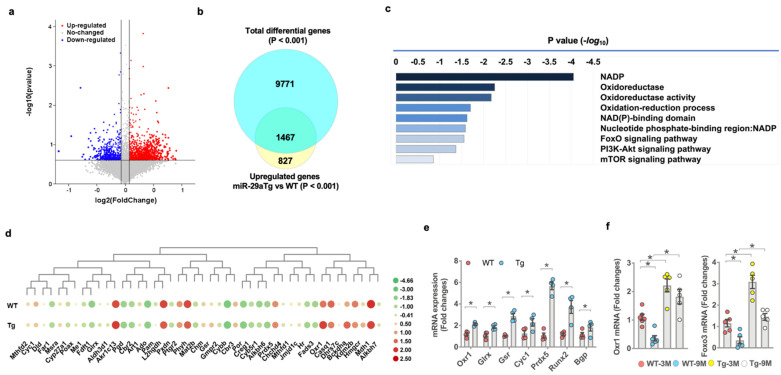
Transcriptomic landscapes of miR-29aTg and wild-type osteoblasts. Volcanic plots showing differential gene expression in osteoblasts between 3-month-old miR-29aTg and WT mice (**a**). Red, blue, and grey spots indicate a gene expression that is upregulated, downregulated, and unaffected, respectively. Venn diagram showing the gene transcripts, which were increased the miR-29aTg group (**b**). Pathway ontology analysis revealing significantly affected biological reactions in the miR-29aTg group (**c**). Heatmap of transcriptomic profile showing 45 gene transcripts in redox reactions (**d**). Transcriptomic profiles of miR-29aTg and wild-type osteoblasts were analyzed in 3 mice. *Orx1*, *Glrx*, *Gsr*, *Cyc1*, *Prdx5*, *Runx2*, and *Bgp* mRNA expression was increased in miR-29aTg osteoblasts, as evidenced in RT-PCR analysis (**e**). Significant difference of investigations (mean ± standard error, *n* = 4 mice) was analyzed using a Student’s *t*-test. Orx1 and FoxO3 loss in bone-marrow mesenchymal cells was compromised in old miR-29aTg mice (**f**). Significant differences (asterisks *) of investigations (*n* = 5 mice) were analyzed using an ANOVA test and a Bonferroni post hoc test.

We characterized transcriptomic landscapes of 3-month-old miR-29aTg and WT osteoblasts using whole-genome microarray analysis (Figure 5a). Of 9971 differentially expressed genes, *miR-29a* upregulated 1467 gene transcription (Figure 5b) related to a plethora of redox reactions and intracellular pathways, including NADP, oxidoreductase, oxidoreduction activity, FoxO, PI3K/Akt, and mTOR signaling pathways, as evidenced in pathway ontology analysis (Figure 5c). *miR-29a* overexpression increased the gene transcription of antioxidant proteins, such as oxidation resistance 1 (Oxr1), glutaredoxin (Glrx), glutathione disulfide reductase (Gsr), cytochrome 1 (cyc1), and peroxiredoxin 5 (Prdx5) (Figure 5d). RT-PCR analysis confirmed significant increases in *Oxr1*, *Glrx*, *Gsr*, *Cyc1,* and *Prdx5* expression and promoted *Runx2* and *Bgp* expression in miR-29aTg osteoblasts (Figure 5e). Oxr1 and FoxO3 are important to counteract oxidative stress or cellular senescence [31,32]. We uncovered that *Oxr1* and *FoxO3* expression of bone-marrow stromal cells was significantly inhibited in old WT bone tissue. *Oxr1* and *FoxO3* loss were improved in old miR-29aTg mice (Figure 5f).

### 3.6. miR-29a Repressed DNMT3b-Mediated Foxo3 Methylation

**Figure 6:**
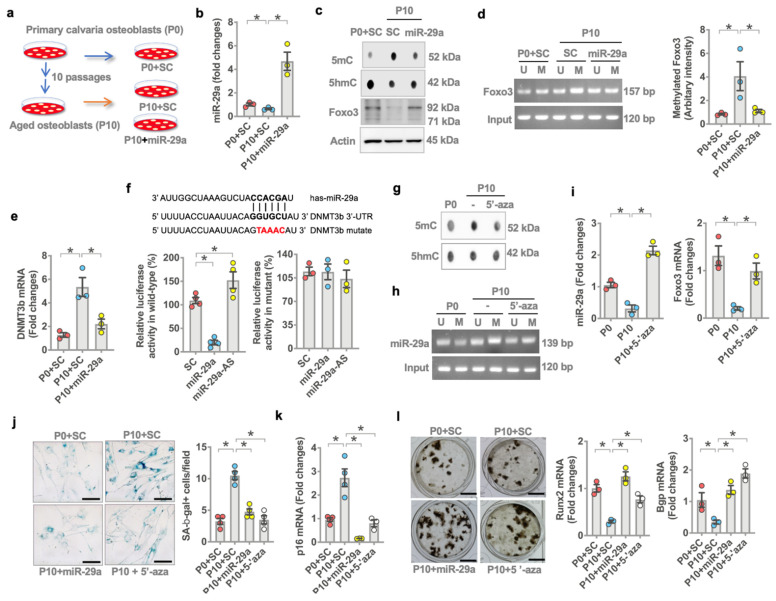
Effects of *miR-29a* on *FoxO3* promoter methylation and *Dnmt3b* expression in aged osteoblasts. Schematic drawing for a model of multiple passages-induced osteoblast aging (**a**). Forced *miR-29a* expression reversed *miR-29a* (**b**) and FoxO3 levels but decreased 5mC levels (**c**) and *FoxO3* promoter methylation (**d**) in multiple-passaged osteoblasts. *miR-29a* attenuated (**e**) *Dnmt3b* expression (**e**) and luciferase reporter activity of 3′-UTR of *Dnmt3b* (**f**). 5-aza decreased 5mC levels (**g**) and *miR-29a* promoter methylation (**h**), reversing *miR-29a* and FoxO3 expression (**i**) in multiple passage osteoblasts. *miR-29a* or 5-aza compromised multiple-passage-induced SA-*β*-gal activity (**j**) and p16 expression (**k**), improving mineralized matrix production and *Runx2* and *Bgp* expression (**l**). Significant differences (asterisks *) of investigations (mean ± standard error, *n* = 3–4 experiments) were analyzed using an ANOVA test and a Bonferroni post hoc test. SC, scramble control; U, unmethylated amplicons; M, methylated amplicons. AS, antisense oligonucleotide; 5-aza, 5′-azacytosine.

The analysis of less DNA methylation and mild *FoxO3* loss in an old miR-29aTg bone microenvironment was used to verify if the microRNA affected *FoxO3* methylation in aged osteoblasts. Multiple passaged osteoblasts (passage 10, P10) (Figure 6a) were incubated as a model of osteoblast aging [26,27,28]. *miR-29a* expression (Figure 6b) and FoxO3 level were reduced upon multiple passages, whereas 5mC levels were increased (Figure 6c). Forced *miR-29a* expression by *miR-29a* mimetic downregulated DNA methylation, reversing FoxO3 signaling in multiple-passaged osteoblasts (Figure 6c). The methylation of CpG dinucleotide around −4057~−2058 bp in the transcription start site of the *FoxO3* promoter (Figure 6d) was significantly upregulated in multiple-passaged cells. *miR-29a* attenuated *FoxO3* promoter hypermethylation in aged osteoblasts (Figure 6d), as is evident from the bisulfite conversion of DNA and methylation-specific PCR analysis.

We investigated the mechanism by which *miR-29a* repressed *FoxO3* methylation in aged osteoblasts. A bioinformatics prediction (http://targetscan.org/vert_72/, accessed on 19 July 2021) shows that DNA methyltransferase 3b (*Dnmt3b*) catalyzes DNA methylation and is a putative target of *miR-29a*. *Dnmt3b* expression was increased in multiple-passaged osteoblasts. This effect was downregulated in *miR-29a* mimetic-treated osteoblasts (Figure 6e). *miR-29a* also reduced luciferase reporter activity of wild-type rather than mutated 3′-untranslated regions (3′-UTR) of *Dnmt3b* (Figure 6f). *miR-29a* interference by antisense oligonucleotide increased the luciferase activity, indicating that *miR-29a* directly disrupted *Dnmt3b* mRNA expression (Figure 6f). Of interest, the inhibition of Dnmt3b activity by 5-aza-2-deoxycytidine (5-aza) attenuated DNA hypermethylation (Figure 6g), downregulating methylation of the CpG island around −788~1912 bp in the transcription start site of *miR-29a* promoter (Figure 6h) and improving *miR-29a* and *Foxo3* expression (Figure 6i) in multiple-passaged osteoblasts. As a result, *miR-29a* or 5-aza downregulated SA-β-gal activity (Figure 6j) and *p16* expression (Figure 6k), preserving mineralized matrix production, *Runx2* and *Bgp* expression (Figure 6l) in multiple-passaged cells.

### 3.7. miR-29a Attenuated Oxidative Stress, Aging and Osteogenic Activity

**Figure 7:**
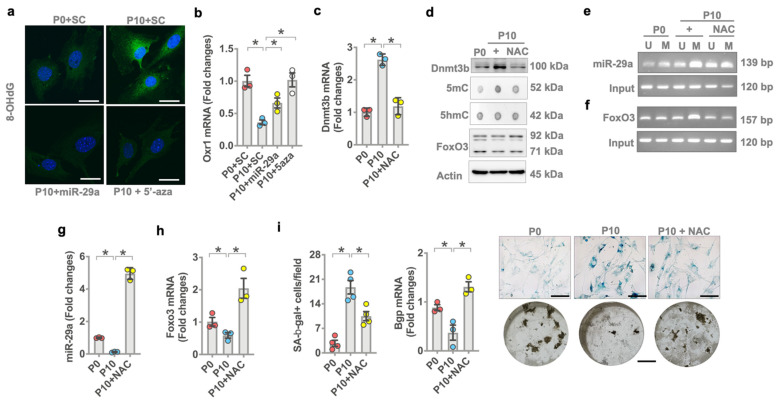
Effects of N-acetylcysteine on *miR-29a* expression and osteoblast senescence. *miR-29a* mimetic or 5-aza attenuated age-induced 8-OHdG immunofluorescence (**a**) (scale bar, 10 μm) and *Orx1* loss (**b**). Antioxidant NAC attenuated *Dnmt3b* mRNA expression (**c**) and 5mC levels (**d**). NAC attenuated age-induced *miR-29a* promoter methylation (**e**) and *FoxO3* promoter methylation (**f**), reversing *miR-29a* (**g**) and *FoxO3* mRNA expression (**h**) in aged osteoblasts to decrease SA-β-gal activity (**i**); (scale bar, 20 μm) and reversed Bgp expression and mineralized nodule formation (**i**). Significant differences (asterisks *) of investigations (mean ± standard error, *n* = 3 experiments) were analyzed using an ANOVA test and a Bonferroni post hoc test. SC, scramble control; U, unmethylated amplicon; M, methylated amplicon. 5-aza, 5′-azacytosine; NAC, N-acetylcysteine.

Of interest, forced *miR-29a* expression or 5-aza also reversed oxidative stress in multiple-passaged cells as was evident from weak 8-OHdG immunofluorescence (Figure 7a) and increased antioxidant protein *Oxr1* expression (Figure 7b). We investigated whether the repression of oxidative stress changed *miR-29a* or *FoxO3* methylation in aged osteoblasts. Antioxidant N-acetylcysteine (NAC) repressed *Dnmt3b* mRNA expression (Figure 7c) and protein levels together with decreased 5mC levels (Figure 7d) in multiple-passaged osteoblasts. NAC also inhibited *miR-29a* promoter methylation (Figure 7e) and *FoxO3* promoter methylation (Figure 7f) to reverse *miR-29a* (Figure 7g) and *FoxO3* mRNA expression (Figure 7h) and protein levels (Figure 7d) and improved SA-β-gal activity, osteocalcin expression, and mineralized nodule formation in multiple-passaged osteoblasts (Figure 7i).

### 3.8. Oxr1 Attenuated Oxidative Stress-Induced miR-29a Loss and Senescence

**Figure 8:**
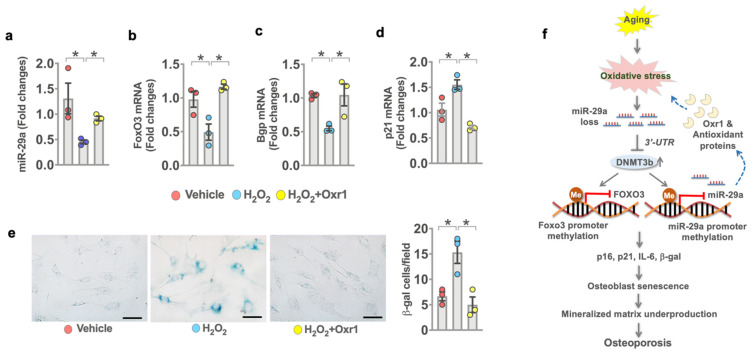
Effects of Oxr1 recombinant protein on *miR-29a* signaling and senescence in MC3T3-E1 osteoblasts. Oxr1 attenuated H_2_O_2_-mediated loss in *miR-29a* (**a**), *FoxO3* (**b**), and *Bgp* (**c**) expression and the inhibited *p21* expression (**d**) of murine osteoblasts. Orx1 reversed H_2_O_2_-induced SA-*β*-gal activity (**e**); scale bar, 15 μm. A significant difference (asterisk *) of investigations (mean ± standard error, *n* = 3 experiments) was analyzed using an ANOVA test and a Bonferroni post hoc test. Schematic graphs showing *miR-29a* protection against oxidative stress, osteoblast senescence, and osteoporosis. *miR-29a* targets Dnmt3b-mediated Foxo3 promoter hypermethylation and increased antioxidant protein Oxr1, compromising age-induced Foxo3 loss, oxidative stress, osteoblast senescence, and osteoporosis development (**f**).

Given that *miR-29a* and Orx1 loss were present in senescent osteoblasts, we investigated what role antioxidant protein Oxr1 may play in *miR-29a* control of osteoblast senescence. Osteoblasts were incubated in H_2_O_2_ as an in vitro model of oxidative stress_2_-induced cellular senescence [29]. Murine MC3T3-E1 osteoblasts were incubated in an osteogenic medium with or without H_2_O_2_ or Oxr1 recombinant protein for 15 days. H_2_O_2_ significantly reduced *miR-29a* (Figure 8a), *FoxO3* (Figure 8c), and *Bgp* expression (Figure 8c), whereas oxidative stress upregulated *p21* expression (Figure 8d) as compared to the vehicle group. Oxr1 recombinant protein reversed H_2_O_2_-induced loss in *miR-29a*, *FoxO3*, and *Bgp* expression and attenuated *p21* expression. Plenty of osteoblasts showed strong SA-β-gal activity upon incubating in H_2_O_2_. Oxr1-treated cells showed weak SA-β-gal staining (Figure 8e).

## 4. Discussion

Osteogenic cell senescence involves postnatal bone homeostasis, microstructure integrity, or osteoporosis development [33]. Programmed senescence in osteogenic progenitor cells sustains long bone development in late pubertal mice [34]; however, senescent osteoblast overburden in a skeletal microenvironment correlates with the development of age [3], chemotherapy [35], and irradiation [36]-induced bone mass loss and architecture damage. Accumulating studies reveal that oxidative stress [37] and the epigenetic pathway [38], including DNA methylation and microRNA signaling, regulate cell fate and metabolism. Little is known about the epigenetic pathway control of oxidative stress in osteoblast aging and bone loss. This study uncovered that *miR-29a* slowed osteoblast senescence, preserving extracellular mineralized matrix production to protect from bone loss in old mice. *miR-29a* attenuated FoxO3 loss and oxidative stress through repressing Dnmt3b-mediated antioxidant protein Oxr1 loss and DNA hypermethylation in aged osteoblasts. Collective evidence conveys a new insight into the antioxidation and anti-aging potential of *miR-29a* against bone loss.

Investigations of clinical specimens uncovered a plethora of reactions, such as *miR-29a* loss, DNA hypermethylation, osteoblast senescence, and oxidative stress in human osteoporosis. Expanding studies show increased *p16* expression [39] or DNA methylome alteration [13] in bone biopsies of human postmenopausal osteoporosis. This analysis prompted us to investigate what role *miR-29a* may play in DNA methylation and oxidative stress in osteoporosis.

We, for the first time, have revealed phenotypes of low bone mass in *miR-29a* knockout mice, suggesting that this molecule was required to maintain skeletal tissue integrity. The adverse effect of *miR-29a* loss on bone homeostasis is also pointed out by a study manifesting that transgenic overexpression of *miR-29-3p* tough decoy in mice inhibits bone mineral accretion and trabecular bone volume [40]. Nine-month-old mice develop osteoporosis, including low bone mass or trabecular volume [26,27,28,29]. In this study, the extent of skeletal deterioration was worsened in 9-month-old miR-29aKO mice. *miR-29a* deletion induced DNA hypermethylation and osteoblast senescence. The impact of *miR-29a* loss on osteoblast fate and bone integrity in this murine model further explained the histological features of senescent osteoblast overburden in human osteoporotic bone.

Striking findings were that old miR-29Tg mice showed fewer osteoporosis signs, including mild MAR loss and osteoclast formation, than age-matched WT mice. *miR-29a* appeared to attenuate excessive bone turnover by preserving the osteogenic differentiation capacity of mesenchymal progenitor cells and reducing *RANKL* expression to downregulate osteoclastic activity. The senescence-associated secretory phenotype, like *IL-6* expression [41], was also repressed in mesenchymal progenitor cells of old miR-29aTg mice. These investigations underpinned the important role of *miR-29a* in delaying age-induced skeletal deterioration.

We revealed that *miR-29a*, at least in part, disrupted the Dnmt3b catalysis of *FoxO3* promoter hypermethylation to compromise FoxO3 loss, reversing senescence and mineralized matrix underproduction in aged osteoblasts. Transcription factor Foxo3 is important to slow cellular aging, promoting tissue homeostasis and function [35]. *FoxO3* interference induces reactive oxygen radical overproduction, inhibiting the osteogenic differentiation capacity of mesenchymal stem cells [42]. The methylation status of the *FoxO3* gene correlates with human tissue aging [43]. In addition, a reciprocal regulation was present in *miR-29a* and Dnmt3b. Dnmt3b inhibition mitigated *miR-29a* promoter methylation, in turn reversing *miR-29a* and FoxO3 functions to drive osteoblasts away from senescence. The inhibition of DNA methyltransferase in bone-forming cells promotes osteogenic activity. *DNMT3b* knockdown or 5-aza-induced DNA hypomethylation promotes the osteogenic differentiation of human [17] and murine [44]. The array of analysis in this study throws a new light upon an epigenetic crosstalk through which microRNA and methyl DNA regulated osteoblast senescence in the development of osteoporosis.

Oxidative stress is a prominent deleterious reaction, dysregulating protein stability or epigenetic pathways to hinder survival or biological activity in senescent cells [45]. Of note, *miR-29a* repressed 8-OHdG formation in aged osteoblasts. A transcriptomic landscape was that *miR-29a* affected redox reactions by promoting a plethora of antioxidant proteins in osteoblasts. Expanding studies reveal the biological roles of Oxr1, Glrx, and Gsr in cellular senescence. For example, forced *Gsr* expression downregulates *Klotho* loss-mediated renal cell aging and dysfunction [46]. Antioxidant protein Oxr1 promotes senolytic activity to sustain the viability of aged fibroblasts. *Oxr1* knockdown aggravates oxidative stress and apoptosis [31]. In this study, Orx1 was advantageous to fend off oxidative stress, FoxO3 loss, and senescence in H_2_O_2_-treated osteoblasts and aged skeleton. Oxidative stress dysregulates epigenetic signatures, affecting the differentiation capacity of mesenchymal progenitor cells [47]. *miR-29* involves the oxidative stress and viability of human mesenchymal stem cells upon *DGCR8* interference [48]. The in vitro analysis of this study consolidated the importance of *miR-29a* and Oxr1 in repressing oxidative stress to downregulate DNA hypermethylation-induced osteoblast dysfunction. In this study, patients in the osteoporosis group were significantly older than the control group. However, the limitation of the experiment for human bone biopsies is the absence of osteoporosis indications in age-matched young patients who required spinal surgery. The possibility cannot be ruled out that ubiquitous *miR-29a* knockout may change other tissues’ integrity or function, which may influence osteoblast function and bone homeostasis. *miR-29a* signaling may affect other DNA methylation enzymes or redox regulators to maintain anabolic activity in aged osteoblasts. The investigations related to the interactions of *miR-29a*, oxidative stress, and epigenetic pathways reveal a new molecular mechanism underlying bone mass loss.

## 5. Conclusions

Taken together, profound evidence revealed that *miR-29a* loss, oxidative stress, and DNA hypermethylation correlated with osteoblast aging in human osteoporosis. *miR-29a* knockout accelerated osteoblast senescence and bone loss. *miR-29a* reversed FoxO3 loss to improve senescence program through targeting Dnmt3b-mediated *FoxO3* methylation and increased antioxidant proteins, including Oxr1, to downregulate oxidative stress and DNA methylation, compromising age-induced osteoblast loss and osteoporosis (Figure 8f). This study highlights a new epigenetic mechanism underlying oxidative stress-mediated osteoblast senescence and senile osteoporosis, as well as the anti-aging and antioxidation effects of *miR-29a*, DNA methylation inhibitor, and Oxr1 on age-induced osteoblast dysfunction and bone loss.

## Data Availability

The data that support the findings of this study are available on request from the corresponding author. The data related to patients’ information are not publicly available due to privacy or ethical restrictions.

## Supplementary Materials

The following are available online at https://www.mdpi.com/article/10.3390/antiox10081248/s1, Table S1: Sequences of primers for RT-PCR and MSP-PCR analysis.
