# Supplementary material for: MicroRNA-29a Mitigates Osteoblast Senescence and Counteracts Bone Loss through Oxidation Resistance-1 Control of FoxO3 Methylation
Source: Antioxidants (Basel). 2021 Aug 4;10(8):1248. doi: 10.3390/antiox10081248 (PMC8389244; doi:10.3390/antiox10081248)
Supplement: Supplementary file 1 [file antioxidants-10-01248-s001.zip › antioxidants-1325257-supplementary.pdf]

Supplementary Table S1

Sequences of primers for RT-PCR and MSP-PCR analysis

Primers for miR-29aKO mice genotyping	
loxP	Forward,5'-ATAGCGGCCGCTCCTCTCACATTGACTGG-3<br>Reverse 5'-GAAGTC GACAGCCCTGAAGTAAGTTC-3’
**Primers for RT-qPCR analysis**	
miR-29a	UCACAGAACCGGUCUCUUU
U6	GTGCTCGCTTCGGCAGCACATATACTAAAATTGGAAC<br>GATACAGAGAAGATTAGCATGGCCCCTGCGCAAGGA<br>TGACACGCAAATTCGTGAAGCGTTCCATATTTT
p16	Forward: 5’-CCGCTGCAGACAGACTGG-3’<br>Reverse: 5’-CTACCTGAATCGGGGTACGA-3’
p21	Forward: 5’-CGGTGGAACTTTGACTTCGT-3’<br>Reverse: 5’-CAGGGCAGAGGAAGTACTGG-3’
IL-6	Forward: 5’-CCACCGGGAACGAAAGAGAA-3’<br>Reverse: 5’- GAGAAGGCAACTGGACCGAA-3’
Foxo3	Forward: 5’-AGCCGTGTACTGTGGAGCTT-3’<br>Reverse: 5’-TCTTGGCGGTATATGGGAAG-3’
Dnmt3b	Forward: 5’-GACGTCGAGCATCATCTTCA-3’<br>Reverse: 5’-ACCCTCCTGATCTCCATCCT-3’
Orx1	Forward: 5’-GGTCTGGAAACAGCCACTAAA-3’<br>Reverse: 5’-GGTGGGAAGAATCCTGGTTATC-3’
Glrx	Forward: 5’-GTAGAGATCGACGGGATGAAAC-3’<br>Reverse: 5’- CCTTCATGTCCTTCCCATAGAG-3’
Gsr	Forward: 5’-ATCCGCCTATGCAGTCTTTAC-3’<br>Reverse: 5’- TCCTCTAACTTGGGTGCTTTG -3’
Cyc1	Forward: 5’-AATCTCGTACGTAACCT-3’<br>Reverse: 5’-TAATTCCGGTAACTTACGTA -3’
Prdx5	Forward: 5’- ATGGTGATAGACAACGGCATAG-3’<br>Reverse: 5’- CAGAGTTGAGAGAGGATGTTGG-3’
Runx2	Forward: 5’-CCAGCAGCACTCCATATCTC-3’<br>Reverse: 5’-CAGCGTCAA CACCATCATTC -3’
Ocn	Forward: 5’-CAAGCAGGGAGGCAATAAGG-3’<br>Reverse: 5’-CGTCAC AAGCAGGGTTAAGC-3’

RANKL	Forward: 5’-CATCGGGTTCCCATAAAG-3’<br>Reverse: 5’- AAAGCAAATGTTGGCGTA-3’
Actin	Forward: 5’-GACGGCCAGGTCATCACTAT-3’<br>Reverse: 5’-CTTCTGCAT CCTGTCAGCA A-3’
**Primers for MSP-PCR analysis**	
Methylated Foxo3 promoter	Forward: 5’-TGATTGTTTATATTTAAAGCGTTGC-3’<br>Reverse: 5’-TATCTACCTAACCTTTTACGACCGA-3’
Unmethylated Foxo3 promoter	Forward: 5’-ATTGTTTATATTTAAAGTGTTGTGT-3’<br>Reverse: 5’-CAACTATCTACCTAACCTTTTACAACCA -3’
Methylated miR-2a promoter	Forward: 5’-GTTGGTAAATAAGCGCGTTAGTC-3’<br>Reverse: 5’-AACGAAAAACTTTAAAAACCTCGA -3’
Unmethylated miR-29a promoter	Forward: 5’-GTGTGTTGGTAAATAAGTGTGTTAGTTG -3’<br>Reverse: 5’-CAAAACAAAAAACTTTAAAAACCTCA -3’
GADPH	Forward: 5’-TACTAGCGGTTTTACGGGCG-3’<br>Reverse: 5’-TCGAACAGGAGGAGCAGAGAGCGA-3’
